# Lack of significant recovery of chloroquine sensitivity in *Plasmodium falciparum* parasites following discontinuance of chloroquine use in Papua New Guinea

**DOI:** 10.1186/s12936-018-2585-x

**Published:** 2018-11-26

**Authors:** Makoto Sekihara, Shin-Ichiro Tachibana, Masato Yamauchi, Shoki Yatsushiro, Steven Tiwara, Naoyuki Fukuda, Mie Ikeda, Toshiyuki Mori, Makoto Hirai, Francis Hombhanje, Toshihiro Mita

**Affiliations:** 1 Department of Tropical Medicine and Parasitology, Juntendo University, Faculty of Medicine, 2-1-1 Hongo, Bunkyo-ku, Tokyo, 113-8421 Japan; 2 Health Technology Research Center, National Institute of Advanced Industrial Science and Technology (AIST), Takamatsu, Japan; 3 Wewak General Hospital, Wewak, East Sepik Province Papua New Guinea; 4 Centre for Health Research & Diagnostics, Divine Word University, P.O. Box 483, Madang, Papua New Guinea

**Keywords:** *Plasmodium falciparum*, Chloroquine, Resistance, Recovery, *pfcrt*, Fitness, Papua New Guinea

## Abstract

**Background:**

Chloroquine treatment for *Plasmodium falciparum* has been discontinued in almost all endemic regions due to the spread of resistant isolates. Reversal of chloroquine susceptibility after chloroquine discontinuation has been reported in dozens of endemic regions. However, this phenomenon has been mostly observed in Africa and is not well documented in other malaria endemic regions. To investigate this, an ex vivo study on susceptibility to chloroquine and lumefantrine was conducted during 2016–2018 in Wewak, Papua New Guinea where chloroquine had been removed from the official malaria treatment regimen in 2010. Genotyping of *pfcrt* and *pfmdr1* was also performed.

**Results:**

In total, 368 patients were enrolled in this study. Average IC_50_ values for chloroquine were 106.6, 80.5, and 87.6 nM in 2016, 2017, and 2018, respectively. These values were not significantly changed from those obtained in 2002/2003 (108 nM). The majority of parasites harboured a *pfcrt* K76T the mutation responsible for chloroquine resistance. However, a significant upward trend was observed in the frequency of the K76 (wild) allele from 2.3% in 2016 to 11.7% in 2018 (*P* = 0.008; Cochran–Armitage trend test).

**Conclusions:**

Eight years of chloroquine withdrawal has not induced a significant recovery of susceptibility in Papua New Guinea. However, an increasing tendency of parasites harbouring chloroquine-susceptible K76 suggests a possibility of resurgence of chloroquine susceptibility in the future.

**Electronic supplementary material:**

The online version of this article (10.1186/s12936-018-2585-x) contains supplementary material, which is available to authorized users.

## Background

Malaria is still one of the three major infectious diseases worldwide with 216 million cases and 445,000 deaths over 100 countries in 2016 [1]. Although new sustainable development goals have proposed to end malaria epidemic by 2030 [2], emergence and spread of drug-resistant parasites could be a major obstacle for this achievement. *Plasmodium falciparum* parasites resistant to artemisinin-based combination therapy (ACT), the current first-line treatment for uncomplicated malaria, have already spread across the Greater Mekong sub-region [3]. However, licensed anti-malarial drugs that possess similar levels of efficacy as artemisinins have not yet been obtained. Under such circumstances, an approach that rotates licensed anti-malarial drugs is suggested to be a potential strategy to combat drug-resistant parasites. Chloroquine is a candidate drug that is potentially applicable to such a strategy. This is because chloroquine-susceptible parasites have outcompeted the resistant parasites and have expanded in the absence of chloroquine selecting pressure [4–6]; subsequently, chloroquine susceptibility has been recovered several years after its discontinuance in many endemic regions, particularly in Africa [7–13]. However, a lack of this phenomenon has been also reported in some African and other endemic regions [14–16]. Therefore, the extent of this reversal across malaria-endemic countries is not fully understood [17].

In Papua New Guinea, since the first report of chloroquine-resistant *P. falciparum* parasite in the 1970s [18], resistant parasites have spread across the area. The clinical efficacy of chloroquine reached unacceptable levels by the mid-1990s [19, 20]. In 2000, a combination regimen of chloroquine or amodiaquine plus sulphadoxine–pyrimethamine was introduced as a first-line treatment for uncomplicated malaria. However, treatment failure of these regimens against *P. falciparum* reached 11–29% during 2003–2005 as assessed at day 28 [21], and 15% during 2005–2007 [22]. In 2010, chloroquine was completely removed from the official treatment regime and artemether plus lumefantrine was officially introduced as a first-line regimen for uncomplicated malaria. Following this discontinuance, change in the average 50% growth inhibitory concentration (IC_50_) to chloroquine was reported as 167 nM during 2005–2007 to 87 nM during 2011–2013 in the Madang Province [23]. However, this IC_50_ value was still much higher than those reported in regions with reversal of chloroquine susceptibility such as Kenya (22.4 nM) [12] and Senegal (34.8 nM) [10]. Additionally, almost all parasites in the Madang study still harboured a chloroquine-resistant allele (K76T mutation) in the *P. falciparum* chloroquine-resistance transporter (*pfcrt*). These results indicate that a complete recovery of chloroquine susceptibility after its withdrawal has not been evidenced in Papua New Guinea and warrants further investigation. An ex vivo study was therefore performed in 2016–2018, 6–8 years after chloroquine withdrawal in Wewak district, East Sepik Province, in which the ex vivo drug susceptibility study was previously conducted during 2002–2003 [24].

## Methods

### Study design and sites

Three cross-section studies for ex vivo malaria drug resistance targeted in symptomatic *P. falciparum*-infected patients were carried out at two clinics (Wirui Urban and Town) in Wewak District of East Sepik Province in Papua New Guinea. The studies began in January and ended in February in 2016 and in 2017 and from February to March in 2018. The average temperature in the studied area is 27.3 °C (min 23.8 °C, max 30.9 °C) with an annual rainfall of approximately 3000 mm. All four species of human malaria parasites were observed with limited seasonal variations between the wet (October to April) and dry (May to September) seasons and were transmitted mainly by *Anopheles farauti, Anopheles punctulatus*, and *Anopheles koliensis* [20, 25, 26].

The Government of Papua New Guinea implemented the first country-wide free distribution of long-lasting insecticidal mosquito nets (LLIN) with financial support from the Global Fund to Fight AIDS, tuberculosis, and malaria between 2005 and 2009 (round 3 grant) and between 2009 and 2013 (round 8 grant) [27]. The average LLIN usage was 55% in 2008 and 2009 [28] and 32.9–67.7% during 2013–2014 [29]. The mean Anopheles man biting rate was 31 bites/person/night, which is much lower than that (83 bites/person/night) observed in the pre-LLIN distribution period [30]. Malaria prevalence has been considerably decreased in all endemic regions; the overall prevalence of all species was 11.1% (2008–2009), 5.1% (2010–2011), and 0.9% (2013–2014) [29]. The current first-line regime includes artemether plus lumefantrine, which was officially introduced in 2010.

Ethical approvals were obtained from the Medical Research Ethical Committee of Juntendo University (No. 13-016) and the Medical Research Advisory Committee of Papua New Guinea National Department of Health (No. 14.22. & 16.41.).

### Patients and blood collection

In both studied clinics, *P. falciparum* infection was screened using a Rapid Diagnosis Test (RDT) (CareStart™ Malaria HRP2/pLDH COMBO Test kit, Access Bio, USA) in patients (> 1 year of age) with symptoms suspected of malaria such as axillary temperature above 37.5 °C or a fever during the previous 24 h, as reported by the family. When a *P. falciparum*-positive result was obtained, patients were enrolled after obtaining informed consent from the patients or guardians. Blood samples were obtained by finger prick (< 2 years, 100–500 μL) or peripheral venipuncture (≥ 2 years, 1 mL) and collected into EDTA-containing tubes and immediately transferred to the central laboratories at Wewak General Hospital. Thick and thin blood smears were prepared and stained with 2% Giemsa for 30 min. Blood samples showing parasitaemia ≥ 0.05% were used for both ex vivo anti-malarial susceptibility assays and molecular analysis. Samples showing parasitaemia < 0.05% were used for molecular analysis alone. For molecular analysis, blood samples were transferred onto chromatography filter paper (ET31CHR; Whatman Limited, Kent, UK) and separated in a plastic bag after drying at a normal temperature and stored at − 20 °C. Species-specific polymerase chain reactions (PCRs) were performed to confirm *P. falciparum* infections, as previously described [31].

### Ex vivo anti-malarial susceptibility assays

Ex vivo assays were performed to determine anti-malarial susceptibility to chloroquine and lumefantrine. After removing the plasma and buffy coat, erythrocyte pellets were washed thrice in complete RPMI1640 medium (Thermo Fisher Scientific Inc., Waltham, MA, USA) with 0.225 mg/mL gentamicin. Washed pellets were suspended in the 2.5% haematocrit of culture medium; RPMI-1640 containing 25 mM HEPES and 2 mM l-glutamine supplemented with 0.25 mg/mL gentamicin and heat-inactivated 10% serum from O blood type Japanese volunteers. Parasite density was adjusted to 0.05% with O type erythrocytes from Japanese volunteers. Next, 100 μL of parasite culture was added to each well of a 96-well culture plate, which was pre-dosed with chloroquine: 0, 25, 50, 100, 200, 400, 800, and 1600 nM or lumefantrine: 0, 1.25, 2.5, 5, 10, 20, 40, and 80 nM. The sample-applied plates were then incubated at 37 °C for 72 h in a gas atmosphere (5% CO_2_, 5% O_2_) created using the AnaeroPack^®^ malaria culture system (Mitsubishi Gas Chemical Company Inc., Tokyo, Japan). Samples were then frozen (− 20 °C overnight) and thawed until complete haemolysis was obtained. Parasite growth was assessed using an enzyme-linked immunosorbent assay (ELISA) that quantifies parasite histidine-rich protein-2 (HRP-2) as reported previously [32]. The effective concentration needed to inhibit *P. falciparum* growth by 50% (IC_50_) was established by non-linear regression using an online ICEstimator software (http://www.antimalarial-icestimator.net) [33].

### Multiplicity of infections (MOIs)

*Plasmodium falciparum* DNA was extracted from a quarter of a blood spot (25 µL) using the QIAamp DNA blood Mini Kit (QIAGEN, Hilden, Germany). MOIs or the number of clones per sample were determined by genotyping of *merozoite surface protein 2* (*msp2*), the gene encoding the highly polymorphic locus MSP2, as reported previously [34]. Briefly, a nested multiplex PCR was performed to amplify 3D7 and/or FC27 family alleles using fluorescence-labelled family-specific primers with Tks Gflex DNA Polymerase (Takara Bio Inc., Japan) in a 10-μL reaction mixture containing 1 μL of DNA template and 0.5 μM of each primer set. The nested PCR products were analysed by 2% agarose gel electrophoresis to select the samples for subsequent capillary electrophoresis analysis. Size variations of nested PCR products were analysed using an Applied Biosystems 3130/3130xl Genetic Analyzer (Life Technologies, Carlsbad, California, USA) and determined with the Peak Scanner software ver2.0 (Thermo Fisher Scientific). If minor peak heights were greater than one-third of the major peak height, these minor peaks were regarded as peaks from minor clones. Samples harbouring two or more alleles were interpreted as multiple-clonal infections.

### Genotyping of *pfcrt* and *pfmdr1*

Polymorphisms at position 72–76 in *pfcrt* and at positions 86, 184, 1034, 1042, and 1246 in *P. falciparum multidrug resistance*-*1* (*pfmdr1*), which are suggested to be associated with resistance to a variety of anti-malarial drugs [35], were determined by direct sequencing. An initial and nested PCR were performed with PrimeSTAR Max DNA Polymerase (Takara Bio Inc., Japan) in a 10-μL reaction mixture containing 1 μL of DNA template and 0.5 μM of each primer set. Excess primers and unincorporated nucleotides of the nested PCR product were enzymatically removed using ExoSAP-IT Kit (Amersham Biosciences, Buckinghamshire, UK) and direct sequencing was performed (96 °C or 1 min, 25 cycles of 96 °C for 30 s, 50 °C for 30 s, and 60 °C for 4 min, and 60 °C for 1 min) using a BigDye Terminator v1.1 cycle sequencing kit on the Applied Biosystems 3130/3130xl Genetic Analyzer (Life Technologies, Carlsbad, California, USA). Samples with minor peaks of at least 50% in height compared to the major peak were considered mixed genotypes.

Allele frequencies (proportion of parasite clones in the parasite population that carry a given allele) of drug-resistance genes were estimated using MalHaploFreq [36], a program that utilizes allele prevalence and MOI data to estimate allele frequencies with a maximum likelihood algorithm using the maximum likelihood methodology.

### Statistical analysis

All statistical analyses were performed using R software (Version 3.3.3). Data was analysed using Chi-square test, Fisher’s exact test, Cochran–Armitage trend test, Jonckheere–Terpstrata test, and Welch’s *t*-test. P-value < 0.05 was considered significant.

## Results

### Enrolled patients

Among a total of 453 patients recruited, 60, 7 and 18 patients were diagnosed as *P. vivax* mono-infection, mixed infection with *P. falciparum* and *P. vivax*, and no malaria by using species-specific PCR and were removed from the enrolment. In total, 368 patients were enrolled for this study at two sampling clinics; 182 at Town clinic and 186 at Wirui Urban clinic. They are separated by about 2 km and nearly all background characteristics of enrolled patients were the same between the clinics (Additional file 1). The only difference was found in the frequency of pre-treated patients, which was significantly higher at Town clinic (11.5%) than that at Wirui clinic (3.2%) (*P *= 0.004, Chi-square test). The number of enrolled patients was similar for each year; 123 in 2016,134 in 2017, and 111 in 2018. Background characteristics of the enrolled patients did not significantly differ among the studied year.

Nearly 40% of the patients were 10 to 19 years old (Table 1). No severe case was enrolled in the study. Median initial parasitaemia was 0.14%, 0.33%, and 0.12% in 2016, 2017, and 2018, respectively. In total, 27 enrolled patients presented a history of ingesting anti-malarial drug(s) within 2 weeks. Artemether alone (n = 12) and chloroquine (n = 10) were the two most used forms of self-medication. These patients were removed from further analysis, resulting in 341 patient samples.

**Table 1:** Characteristics of enrolled patients

Characteristics	2016 (N = 123)	2017 (N = 134)	2018 (N = 111)
Sampling clinics; n (%)			
Wirui urban	89 (72.4)	40 (29.9)	57 (51.4)
Town	34 (27.6)	94 (70.1)	54 (49.6)
Age; n (%)			
0–4	2 (1.6)	5 (3.7)	3 (2.7)
5–9	13 (10.6)	19 (14.2)	19 (17.1)
10–19	43 (35)	55 (41)	44 (39.6)
20–29	28 (22.8)	32 (23.9)	24 (21.6)
30–39	15 (12.2)	9 (6.7)	8 (7.2)
40–49	11 (8.9)	6 (4.5)	7 (6.3)
50	9 (7.3)	7 (5.2)	5 (4.5)
Unknown	2 (1.6)	1 (0.7)	1 (0.9)
Average	23.9	19.8	20.5
Sex; n (%)			
Male	53 (43.1)	57 (42.5)	57 (51.4)
Female	69 (56.1)	76 (56.7)	53 (47.7)
Unknown	1 (0.8)	1 (0.8)	1 (0.9)
Pretreatment; n (%)			
Artemether	3 (2.4)	4 (3.0)	5 (4.5)
Artemether + lumefantrine	0 (0)	2 (1.5)	1 (0.9)
Artemether + lumefantrine + primaquine	0 (0)	1 (0.8)	0 (0)
Chloroquine	2 (1.6)	5 (3.7)	3 (2.7)
Primaquine	1 (0.8)	0 (0)	0 (0)
Parasitemia; (%)			
Median (IQR)	0.14% (0.02%, 0.52%)	0.33% (0.1%, 0.88%)	0.12% (0.1%, 0.57%)
MOI; n			
1	98	113	ND
2	13	9	ND
3	1	0	ND
Mean	1.13	1.07	ND

*IQR* interquartile range, *ND* not determined

### Ex vivo susceptibility to chloroquine and lumefantrine

Among the 341 patient samples, 113 were excluded from the ex vivo drug-susceptibility assay because of low (< 0.05%) parasitaemia (n = 80) and lack of blood volume (n = 33); finally, blood samples from 228 patients were used in the ex vivo drug-susceptibility assay (62 in 2016, 101 in 2017, and 65 in 2018). Among these, interpretable ex vivo drug susceptibility data that fulfilled the criteria for core analysis [37] were obtained in 174 assays for chloroquine. However, as high confidence on the estimated IC_50_ is tremendously important for this study, 36 results showing a ratio of high to low 95% confidence intervals for IC_50_ > 2 were further excluded [33]. This resulted in a total of 138 estimated IC_50_ values that almost completely fit with the inhibitory sigmoid Emax model (Table 2). Average IC_50_ values to chloroquine were 106.6, 80.5, and 87.6 nM in 2016, 2017, and 2018, respectively. Although these values were slightly lower than those obtained in the previous study during 2002/2003 in the same study area (IC_50_ = 108 nM) [24], these differences were not statistically significant. Accordingly, a decreasing trend was not found in the average IC_50_ values during 2016–2018 (Jonckheere–Terpstrata test).

**Table 2:** Ex vivo susceptibility of clinical parasites of *P. falciparum* in Papua New Guinea

Drug	No	Mean IC50 (95% CI)		
		2016	2017	2018
Chloroquine	138	106.6 nM (79.4 nM, 133.9 nM)	80.5 nM (68.6 nM, 92.3 nM)	87.6 nM (72.6 nM, 102.5 nM)
Lumefantrine	74	–	4.6 (4.05 nM, 5.16 nM)	–

*CI* confidence intervals

Ex vivo drug susceptibility assay for lumefantrine was performed in 99 patients in 2017, producing 85 interpretable results. A total of 11 cases that showed a ratio of high to low 95% confidence intervals for IC_50_ > 2 were further removed, which resulted in a total of 74 final results. The mean IC_50_ for lumefantrine was 4.6 nM.

### Prevalence and frequencies of polymorphisms in *pfcrt* and *pfmdr1*

The prevalence of specific mutations in *pfcrt* and *pfmdr1* was determined (Additional file 2). Allele frequencies of *pfcrt* and *pfmdr1* were also estimated based on the prevalence of these alleles and Multiplicity of infections (MOIs) using MalHaploFreq [36] (Table 3). In 2018, because blood samples showing mixed alleles (ex, K76 + K76T in *pfcrt*) were not observed in both *pfcrt* and *pfmdr1*, allele frequencies were identical to allele prevalence and thus, MOIs were not determined in 2018. The maximum MOI detected was 3 and was observed in one sample (Table 1). Mean MOIs in 2017 (1.07) were slightly lower than those in 2016 (1.13), both of which were similar or slightly lower than those previously observed in highland areas [38].

**Table 3:** Allele frequencies in *pfcrt* and *pfmdr1*

	2016	2017	2018
	% CI^b^(%)	% CI(%)	%
*Pfcrt* ^a^			
CVMNK	2.3 (0.5–6.1)	10.4 (5.9–16.5)	11.7
SVMNT	97.7 (93.9–99.5)	89.6 (83.5–94.1)	88.3
*Pfmdr1*			
N86	58.7 (50–67.2)	71.2 (63.2–78.5)	73.3
N86Y	41.3 (32.8–50)	28.8 (21.5–36.8)	26.7
Y184	79.8 (72.2–86.3)	71.2 (63.2–78.5)	84.4
Y184F	20.2 (13.7–27.8)	28.8 (21.5–36.8)	15.6
S1034	100	100	100
S1034C	0	0	0
N1042	92 (85–96.6)	88.6 (81.4–93.9)	89
N1042D	8 (3.4–15)	11.4 (6.1–18.6)	11
D1246	100	100	100
D1246Y	0	0	0

^a^Amino acids at positions 72–76, mutation underlined

^b^95% confidential interval

Sequence analysis of codon 72–76 in *pfcrt* revealed two haplotypes, wild-type (CVMNK) and a mutant (SVMNT) (amino acids at positions 72–76, mutation underlined). The frequency of K76 depicted a significant upward trend: 2.3% in 2016, 10.4% in 2017, and 11.7% in 2018 (*P* = 0.008; Cochran–Armitage trend test). Similarly, a significant increase in the N86 allele in *pfmdr1* was observed: 58.7% in 2016, 71.2% in 2017, and 73.3% in 2018 (*P* = 0.006; Cochran–Armitage trend test). Meanwhile, no significant difference was found at position 184 and 1042 in *pfmdr1*. All isolates possessed a wild-type allele at position 1034 and 1246 in *pfmdr1*.

### Association between ex vivo IC_50_ values for chloroquine and lumefantrine, and mutations in *pfcrt* and *pfmdr1*

**Fig. 1:**
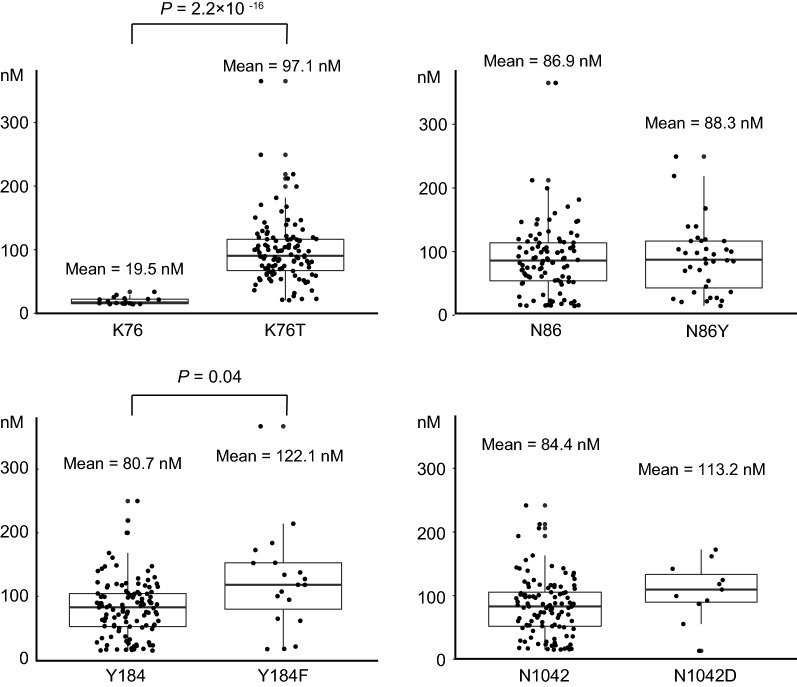
Association between IC_50_ values for chloroquine and polymorphisms in *pfcrt* and *pfmdr1*. Statistical significance was calculated using Welch’s *t*-test

**Fig. 2:**
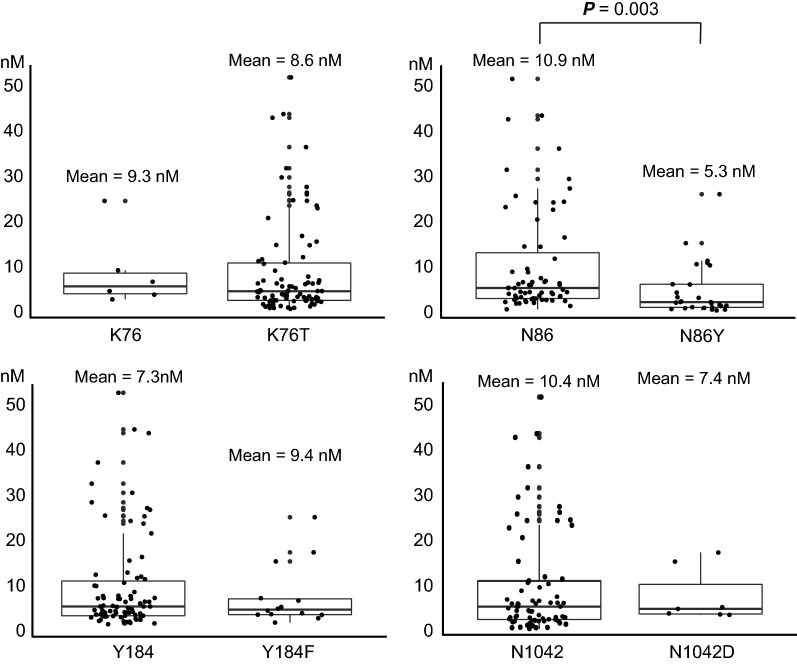
Association between IC_50_ values for lumefantrine and polymorphisms in *pfcrt* and *pfmdr1.* Statistical significance was calculated using Welch’s *t*-test

Parasites harbouring the *pfcrt* K76T mutation depicted significantly higher IC_50_ values for chloroquine (97.1 nM) than those harbouring K76 (19.5 nM) (*P *= 2.2 × 10^−16^, Welch *t*-test) (Fig. 1). In *pfmdr1*, the N86Y mutation was significantly associated with lower IC_50_ values for lumefantrine (5.3 nM in N86Y vs. 10.9 nM in N86, *P* = 0.003, Welch *t*-test) (Fig. 2). Parasites harbouring a Y184F mutation showed significantly higher IC_50_ values for chloroquine (122.1 nM) than those with Y184 (80.7 nM) (*P* = 0.04, Welch *t*-test).

To investigate the potential effect of Y184F in the augmentation of chloroquine resistance in K76T harbouring parasites, average IC_50_ values were compared between Y184 and Y184F in parasites with the *pfcrt* K76T mutation (Additional file 3). The Y184F harbouring parasites displayed significantly higher IC_50_ values (141.4 nM) than those of the Y184 harbouring parasites (90.2 nM) (*P *= 0.02, Welch *t*-test), suggesting that the Y184F mutation may augment the level of chloroquine resistance in *P. falciparum* parasites harbouring K76T in *pfcrt*.

## Discussion

There are dozens of epidemiological studies showing that chloroquine-susceptible parasites replace resistant parasites in the absence of chloroquine selection [7–13]. However, the present analysis revealed a lack of substantial recovery of chloroquine susceptibility at 6–8 years after the withdrawal of chloroquine in Papua New Guinea.

In nearly all endemic regions where chloroquine-sensitive parasites re-emerged, reduction of parasites harbouring a K76T mutation in *pfcrt* played a pivotal role towards this phenomenon [7–10, 12], though some exclusive regions have been reported, such as French Guiana [39]. This is because the K76T mutation imposes some fitness cost to the parasites [40–43]. A reverse genetic study evidenced that introduction of K76T into chloroquine-susceptible clones induced a reduction in the growth rate [41, 42]. One suggested mechanism for this is that K76T harbouring parasites show functional impairment of haemoglobin digestion, which subsequently reduces the supply of amino acids required for parasite growth [41]. Fitness reduction of chloroquine-resistant parasites was also reported in mosquito stages; K76T-bearing parasites were less selected than K76-bearing parasites in *Anopheles arabiensis* [40]. Because of these disadvantages, K76T-harbouring parasites have been outcompeted by K76-harbouring parasites in the absence of chloroquine pressure [4, 6]. In this study, *pfcrt* K76T harbouring parasites showed a significantly higher IC_50_s than those in *pfcrt* K76 harbouring parasites. This observation is same as those observed in African endemic regions [11, 12], suggesting that an associated mechanism of chloroquine resistance would be common in parasites in Africa and Papua New Guinea. However, the majority of parasites still harboured the K76T allele and recovery of chloroquine susceptibility has not been observed even after withdrawal of chloroquine use. These observations suggest that genetic change(s) other than K76T in *pfcrt* and/or other unknown gene(s) compensate the fitness cost imposed by K76T and may explain the reason why chloroquine susceptibility is not returning at the same rate in Africa. It could be also possible to conjecture that, in Africa, there are some particular K76-harbouring parasites which have some stronger fitness advantage than K76-harbouring parasites in Papua New Guinea.

It has been suggested that amino acid differences flanking K76T affect the fitness disadvantage imposed by K76T [42]. In the natural parasite population, there are two major mutant haplotypes constructed by five amino acids at positions 72–76: CVIET and SVMNT [42, 44]. In Papua New Guinea, nearly all mutant parasites harboured a SVMNT haplotype, and the CVIET haplotype was also observed with extremely low prevalence [45, 46]. A transfection study has reported that a SVMNT introduced isolate depicted lower growth rates than a wild-type (CVMNK) isolate, but better growth rate than the CVIET introduced isolate [42]. A quick repopulation of K76-harbouring parasites after chloroquine discontinuance has mostly been observed in the CVIET haplotype regions. Therefore, the fact that all *pfcrt* mutants harboured a SVMNT haplotype may partly explain the persistent high prevalence of K76T in this study region.

However, it is striking that K76-harbouring parasites significantly increased during 2016–2018. This is the first study to show the potential repopulation of K76 harbouring parasites after chloroquine withdrawal in a SVMNT prevalent region. Despite a significant increase, the majority of parasites still harboured the K76T allele. Many environmental, population genetic, and parasitological factors potentially affect the rate of repopulation of susceptible parasites after chloroquine discontinuance [5, 47, 48]. The frequency of susceptible parasites in the parasite population when chloroquine pressure was removed is one such important factor. Historically, the K76T allele had already become predominant or was nearly fixed by the late 1990s in many endemic regions in Papua New Guinea [45, 49–51]. Accordingly, the K76T prevalence in our study region reached around 95% during 2002–2003 [24]. Considering the strong selection pressure posed by the use of chloroquine for the treatment of uncomplicated malaria before 2010, an extremely low frequency of susceptible parasites is expected at the time when chloroquine was withdrawn. Therefore, it is considered that the observed high proportion of K76T harbouring parasites may be partly explained by the presumed extremely low initial proportion of K76-harbouring parasites.

A requirement for secondary determinants has been suggested for the augmentation of chloroquine resistance [52–54]. One such candidate gene is *pfmdr1* [35, 53]. In the present study, parasites with Y184F mutation displayed a significantly higher IC_50_ for chloroquine compared to those with Y184. This association was also found in parasites bearing *pfcrt*-K76T, suggesting that Y184F confers an additional factor for decreased chloroquine susceptibility in our study area. However, a previous reverse genetic study reported that an allele change from Y184 to Y184F conferred only a slight decrease in chloroquine susceptibility in a laboratory clone harbouring *pfcrt*-SVMNT [55]. One possible explanation for this discrepancy is that genetic background could influence the role of the Y184F mutation on the augmentation of chloroquine resistance. The parasite clone used in the study by Veiga et al., was a KC5 clone, a progeny of the genetic cross between 7G8 (Brazil) and GB4 (Ghana) parasites [56].

Persistence of chloroquine-selecting pressure potentially interferes the recovery of chloroquine-sensitive parasites. In Lagos, Nigeria where chloroquine was still widely used even after the introduction of ACT, *P. falciparum* parasites harbouring a K76T mutation continued to be highly prevalent [57]. In Papua New Guinea, however, ACT has been used as a first-line treatment for all malaria species including *P. vivax*. Chloroquine has not been included in the official malaria-treatment regimen. However, although no stock of chloroquine in clinics and hospitals was confirmed in the studied area, chloroquine was still sold at two private pharmacies with a cheaper price than other anti-malarial drugs throughout the study period. Indeed, 2–4% of enrolled patients used chloroquine before visiting the clinics in this study. These observations indicate that chloroquine is still in use by some patients, which could play some role in a result of lack of complete withdrawal of chloroquine.

For lumefantrine, our average IC_50_ values (4.6 nM) were higher than those (1.5 nM) reported in Madang district during 2011–2013 [23]. The N86 allele frequencies in our study (59–74%) were also much higher than those in the Madang study (< 10%). In our study, a significant association was detected between higher IC_50_ values for lumefantrine and the N86 allele in *pfmdr1*. This is consistent with the previous transfection study in which an allelic change from N86Y to N86 resulted in a three to fourfold increase in the IC_50_ for lumefantrine [55]. A recent meta-analysis has also shown that patients infected with parasites harbouring N86 had a fivefold risk of recrudescence in following artemether/lumefantrine treatment compared to those infected with parasites harbouring N86Y [58]. The observed lower lumefantrine susceptibility and higher *pfmdr1*-N86 prevalence than that in the previous observation [23] may raise the possibility of a decreasing trend of lumefantrine susceptibility in Papua New Guinea.

## Conclusions

The present analysis provides molecular and ex vivo evidence for the absence of significant recovery of chloroquine susceptibility after 8 years of chloroquine withdrawal. On the other hand, this study also exhibits a significant increase in parasites harbouring K76 during the study period, albeit still in a small portion of the parasite population. It is well recognized that at the early phase when more fit strains are in a small portion, stochastic reasons rather than selective advantages play an important role in the increase of these strains [59]. Once these strains reach a sufficiently large population, selective advantage considerably affects the expansion of these more fit strains [59]. As such, current study hints at a reversal of chloroquine susceptibility in the future and warrants further continuous molecular epidemiological and phenotypic assessment of natural parasites in Papua New Guinea.

## Additional files

**Additional file 1.** Characteristics of enrolled patients at each sampling site.

**Additional file 2.** Allele prevalence in *pfcrt* and *pfmdr1*.

**Additional file 3.** Association between IC_50_ values for chloroquine and polymorphisms at position 184 in *pfmdr1* in the parasites with the *pfcrt* K76T mutation.

## Abbreviations

ACT: artemisinin-based combination therapy
ELISA: enzyme-linked immunosorbent assay
HRP-2: histidine-rich protein-2
IC50: 50% growth inhibitory concentration
LLIN: long-lasting insecticidal mosquito net
MOI: multiplicity of infection
MSP2: merozoite surface protein 2
RDT: rapid diagnosis test

## References

[1] WHO (2017). World malaria report 2017.

[2] WHO (2015). Global technical strategy for malaria 2016–2030.

[3] Ashley EA, Dhorda M, Fairhurst RM, Amaratunga C, Lim P, Suon S (2014). Spread of artemisinin resistance in *Plasmodium falciparum* malaria. N Engl J Med.

[4] Mita T, Kaneko A, Lum JK, Zungu IL, Tsukahara T, Eto H (2004). Expansion of wild type allele rather than back mutation in pfcrt explains the recent recovery of chloroquine sensitivity of *Plasmodium falciparum* in Malawi. Mol Biochem Parasitol.

[5] Hastings IM, Donnelly MJ (2005). The impact of antimalarial drug resistance mutations on parasite fitness, and its implications for the evolution of resistance. Drug Resist Updat.

[6] Laufer MK, Takala-Harrison S, Dzinjalamala FK, Stine OC, Taylor TE, Plowe CV (2010). Return of chloroquine-susceptible falciparum malaria in Malawi was a reexpansion of diverse susceptible parasites. J Infect Dis.

[7] Mita T, Kaneko A, Lum JK, Bwijo B, Takechi M, Zungu IL (2003). Recovery of chloroquine sensitivity and low prevalence of the *Plasmodium falciparum* chloroquine resistance transporter gene mutation K76T following the discontinuance of chloroquine use in Malawi. Am J Trop Med Hyg.

[8] Kublin JG, Cortese JF, Njunju EM, Mukadam RA, Wirima JJ, Kazembe PN (2003). Reemergence of chloroquine-sensitive *Plasmodium falciparum* malaria after cessation of chloroquine use in Malawi. J Infect Dis.

[9] Laufer MK, Thesing PC, Eddington ND, Masonga R, Dzinjalamala FK, Takala SL (2006). Return of chloroquine antimalarial efficacy in Malawi. N Engl J Med.

[10] Fall B, Diawara S, Sow K, Baret E, Diatta B, Fall KB (2011). Ex vivo susceptibility of *Plasmodium falciparum* isolates from Dakar, Senegal, to seven standard anti-malarial drugs. Malar J.

[11] Eyase FL, Akala HM, Ingasia L, Cheruiyot A, Omondi A, Okudo C (2013). The role of Pfmdr1 and Pfcrt in changing chloroquine, amodiaquine, mefloquine and lumefantrine susceptibility in western-Kenya *P. falciparum* samples during 2008–2011. PLoS ONE.

[12] Lucchi NW, Komino F, Okoth SA, Goldman I, Onyona P, Wiegand RE (2015). In vitro and molecular surveillance for antimalarial drug resistance in *Plasmodium falciparum* parasites in Western Kenya reveals sustained artemisinin sensitivity and increased chloroquine sensitivity. Antimicrob Agents Chemother.

[13] Mbaye A, Dieye B, Ndiaye YD, Bei AK, Muna A, Deme AB (2016). Selection of N86F184D1246 haplotype of *pfmrd1* gene by artemether–lumefantrine drug pressure on *Plasmodium falciparum* populations in Senegal. Malar J..

[14] Ogouyèmi-Hounto A, Ndam NT, Gazard DK, d’Almeida S, Koussihoude L, Ollo E (2013). Prevalence of the molecular marker of *Plasmodium falciparum* resistance to chloroquine and sulphadoxine/pyrimethamine in Benin seven years after the change of malaria treatment policy. Malar J..

[15] Alam MS, Ley B, Nima MK, Johora FT, Hossain ME, Thriemer K (2017). Molecular analysis demonstrates high prevalence of chloroquine resistance but no evidence of artemisinin resistance in *Plasmodium falciparum* in the Chittagong Hill Tracts of Bangladesh. Malar J..

[16] Das S, Tripathy S, Chattopadhayay S, Das B, Kar Mahapatra S, Hati AK (2017). Progressive increase in point mutations associates chloroquine resistance: even after withdrawal of chloroquine use in India. Int J Parasitol Drugs Drug Resist.

[17] Ocan M, Akena D, Nsobya S, Kamya MR, Senono R, Kinengyere AA (2018). Prevalence of chloroquine resistance alleles among *Plasmodium falciparum* parasites in countries affected by malaria disease since change of treatment policy: a systematic review protocol. Syst Rev.

[18] Grimmond TR, Donovan KO, Riley ID (1976). Chloroquine resistant malaria in Papua New Guinea. PNG Med J..

[19] Al-Yaman F, Genton B, Mokela D, Narara A, Raiko A, Alpers MP (1996). Resistance of *Plasmodium falciparum* malaria to amodiaquine, chloroquine and quinine in the Madang Province of Papua New Guinea, 1990–1993. PNG Med J..

[20] Muller I, Bockarie M, Alpers M, Smith T (2003). The epidemiology of malaria in Papua New Guinea. Trends Parasitol..

[21] Marfurt J, Mueller I, Sie A, Maku P, Goroti M, Reeder JC (2007). Low efficacy of amodiaquine or chloroquine plus sulfadoxine–pyrimethamine against *Plasmodium falciparum* and *P. vivax* malaria in Papua New Guinea. Am J Trop Med Hyg.

[22] Karunajeewa HA, Mueller I, Senn M, Lin E, Law I, Gomorrai PS (2008). A trial of combination antimalarial therapies in children from Papua New Guinea. N Engl J Med.

[23] Koleala T, Karl S, Laman M, Moore BR, Benjamin J, Barnadas C (2015). Temporal changes in *Plasmodium falciparum* anti-malarial drug sensitivity in vitro and resistance-associated genetic mutations in isolates from Papua New Guinea. Malar J..

[24] Mita T, Kaneko A, Hombhanje F, Hwaihwanje I, Takahashi N, Osawa H (2006). Role of pfmdr1 mutations on chloroquine resistance in *Plasmodium falciparum* isolates with pfcrt K76T from Papua New Guinea. Acta Trop.

[25] Schultz L, Wapling J, Mueller I, Ntsuke PO, Senn N, Nale J (2010). Multilocus haplotypes reveal variable levels of diversity and population structure of *Plasmodium falciparum* in Papua New Guinea, a region of intense perennial transmission. Malar J.

[26] Barry AE, Schultz L, Senn N, Nale J, Kiniboro B, Siba PM (2013). High levels of genetic diversity of *Plasmodium falciparum* populations in Papua New Guinea despite variable infection prevalence. Am J Trop Med Hyg.

[27] Hetzel MW, Pulford J, Maraga S, Barnadas C, Reimer LJ, Tavul L (2014). Evaluation of the global fund-supported national malaria control program in Papua New Guinea, 2009–2014. PNG Med J..

[28] Hetzel MW, Gideon G, Lote N, Makita L, Siba PM, Mueller I (2012). Ownership and usage of mosquito nets after four years of large-scale free distribution in Papua New Guinea. Malar J.

[29] Hetzel MW, Pulford J, Ura Y, Jamea-Maiasa S, Tandrapah A, Tarongka N (2017). Insecticide-treated nets and malaria prevalence, Papua New Guinea, 2008–2014. Bull World Health Organ.

[30] Hetzel MW, Reimer LJ, Gideon G, Koimbu G, Barnadas C, Makita L (2016). Changes in malaria burden and transmission in sentinel sites after the roll-out of long-lasting insecticidal nets in Papua New Guinea. Parasit Vectors..

[31] Rubio JM, Benito A, Roche J, Berzosa PJ, Garcia ML, Mico M (1999). Semi-nested, multiplex polymerase chain reaction for detection of human malaria parasites and evidence of *Plasmodium vivax* infection in Equatorial Guinea. Am J Trop Med Hyg.

[32] Noedl H, Wernsdorfer WH, Miller RS, Wongsrichanalai C (2002). Histidine-rich protein II: a novel approach to malaria drug sensitivity testing. Antimicrob Agents Chemother.

[33] Le Nagard H, Vincent C, Mentre F, Le Bras J (2011). Online analysis of in vitro resistance to antimalarial drugs through nonlinear regression. Comput Methods Programs Biomed.

[34] Falk N, Maire N, Sama W, Owusu-Agyei S, Smith T, Beck HP (2006). Comparison of PCR-RFLP and Genescan-based genotyping for analyzing infection dynamics of *Plasmodium falciparum*. Am J Trop Med Hyg.

[35] Foote SJ, Kyle DE, Martin RK, Oduola AM, Forsyth K, Kemp DJ (1990). Several alleles of the multidrug-resistance gene are closely linked to chloroquine resistance in *Plasmodium falciparum*. Nature..

[36] Hastings IM, Smith TA (2008). MalHaploFreq: a computer programme for estimating malaria haplotype frequencies from blood samples. Malar J..

[37] Worldwide Antimalarial Resistance Network. WWARN’s in vitro analysis and reporting tool (IVART) http://www.wwarn.org/tools-resources/toolkit/analyse/ivart/ivart-methodology. Accessed 20 Nov 2018.

[38] Fola AA, Harrison GLA, Hazairin MH, Barnadas C, Hetzel MW, Iga J (2017). Higher complexity of infection and genetic diversity of *Plasmodium viva*x than *Plasmodium falciparum* across all malaria transmission zones of Papua New Guinea. Am J Trop Med Hyg..

[39] Pelleau S, Moss EL, Dhingra SK, Volney B, Casteras J, Gabryszewski SJ (2015). Adaptive evolution of malaria parasites in French Guiana: reversal of chloroquine resistance by acquisition of a mutation in pfcrt. Proc Natl Acad Sci USA.

[40] Mharakurwa S, Sialumano M, Liu K, Scott A, Thuma P (2013). Selection for chloroquine-sensitive *Plasmodium falciparum* by wild *Anopheles arabiensis* in Southern Zambia. Malar J..

[41] Lewis IA, Wacker M, Olszewski KL, Cobbold SA, Baska KS, Tan A (2014). Metabolic QTL analysis links chloroquine resistance in *Plasmodium falciparum* to impaired hemoglobin catabolism. PLoS Genet..

[42] Petersen I, Gabryszewski SJ, Johnston GL, Dhingra SK, Ecker A, Lewis RE (2015). Balancing drug resistance and growth rates via compensatory mutations in the *Plasmodium falciparum* chloroquine resistance transporter. Mol Microbiol..

[43] Gabryszewski SJ, Dhingra SK, Combrinck JM, Lewis IA, Callaghan PS, Hassett MR (2016). Evolution of fitness cost-neutral mutant PfCRT conferring *P. falciparum* 4-aminoquinoline drug resistance is accompanied by altered parasite metabolism and digestive vacuole physiology. PLoS Pathog..

[44] Sa JM, Twu O (2010). Protecting the malaria drug arsenal: halting the rise and spread of amodiaquine resistance by monitoring the PfCRT SVMNT type. Malar J.

[45] DaRe JT, Mehlotra RK, Michon P, Mueller I, Reeder J, Sharma YD (2007). Microsatellite polymorphism within pfcrt provides evidence of continuing evolution of chloroquine-resistant alleles in Papua New Guinea. Malar J..

[46] Barnadas C, Timinao L, Javati S, Iga J, Malau E, Koepfli C (2015). Significant geographical differences in prevalence of mutations associated with *Plasmodium falciparum* and *Plasmodium vivax* drug resistance in two regions from Papua New Guinea. Malar J..

[47] Mackinnon MJ, Marsh K (2010). The selection landscape of malaria parasites. Science..

[48] Rosenthal PJ (2013). The interplay between drug resistance and fitness in malaria parasites. Mol Microbiol..

[49] Mehlotra RK, Fujioka H, Roepe PD, Janneh O, Ursos LM, Jacobs-Lorena V (2001). Evolution of a unique *Plasmodium falciparum* chloroquine-resistance phenotype in association with pfcrt polymorphism in Papua New Guinea and South America. Proc Natl Acad Sci USA.

[50] Mehlotra RK, Mattera G, Bhatia K, Reeder JC, Stoneking M, Zimmerman PA (2005). Insight into the early spread of chloroquine-resistant *Plasmodium falciparum* infections in Papua New Guinea. J Infect Dis..

[51] Nsanzabana C, Hastings IM, Marfurt J, Muller I, Baea K, Rare L (2010). Quantifying the evolution and impact of antimalarial drug resistance: drug use, spread of resistance, and drug failure over a 12-year period in Papua New Guinea. J Infect Dis..

[52] Mu J, Ferdig MT, Feng X, Joy DA, Duan J, Furuya T (2003). Multiple transporters associated with malaria parasite responses to chloroquine and quinine. Mol Microbiol..

[53] Sa JM, Twu O, Hayton K, Reyes S, Fay MP, Ringwald P (2009). Geographic patterns of *Plasmodium falciparum* drug resistance distinguished by differential responses to amodiaquine and chloroquine. Proc Natl Acad Sci USA.

[54] Patel JJ, Thacker D, Tan JC, Pleeter P, Checkley L, Gonzales JM (2010). Chloroquine susceptibility and reversibility in a *Plasmodium falciparum* genetic cross. Mol Microbiol..

[55] Veiga MI, Dhingra SK, Henrich PP, Straimer J, Gnadig N, Uhlemann AC (2016). Globally prevalent PfMDR1 mutations modulate *Plasmodium falciparum* susceptibility to artemisinin-based combination therapies. Nature Commun.

[56] Hayton K, Gaur D, Liu A, Takahashi J, Henschen B, Singh S (2008). Erythrocyte binding protein PfRH5 polymorphisms determine species-specific pathways of *Plasmodium falciparum* invasion. Cell Host Microbe..

[57] Oladipo OO, Wellington OA, Sutherland CJ (2015). Persistence of chloroquine-resistant haplotypes of *Plasmodium falciparum* in children with uncomplicated malaria in Lagos, Nigeria, four years after change of chloroquine as first-line antimalarial medicine. Diagn Pathol..

[58] Venkatesan M, Gadalla NB, Stepniewska K, Dahal P, Nsanzabana C, Moriera C (2014). Polymorphisms in *Plasmodium falciparum* chloroquine resistance transporter and multidrug resistance 1 genes: parasite risk factors that affect treatment outcomes for *P. falciparum* malaria after artemether–lumefantrine and artesunate–amodiaquine. Am J Trop Med Hyg..

[59] zur Wiesch PA, Kouyos R, Engelstädter J, Regoes RR, Bonhoeffer S (2011). Population biological principles of drug-resistance evolution in infectious diseases. Lancet Infect Dis..

