# Supplementary material for: Lack of significant recovery of chloroquine sensitivity in Plasmodium falciparum parasites following discontinuance of chloroquine use in Papua New Guinea
Source: Malar J. 2018 Nov 26;17:434. doi: 10.1186/s12936-018-2585-x (PMC6260888; doi:10.1186/s12936-018-2585-x)
Supplement: Supplementary file 1 — Additional file 1. Characteristics of enrolled patients at each sampling site. [file 12936_2018_2585_MOESM1_ESM.pdf]

Additional file 1. Characteristics of enrolled patients at each sampling site

	2016 (N=123)		2017 (N=134)		2018 (N=111)	
	Wirui Urban	Town	Wirui Urban	Town	Wirui Urban	Town
Number of patients; n	89	34	40	94	57	54
Age; n						
0-4	1	1	2	3	1	2
5-9	8	5	6	13	8	11
10-19	34	9	19	36	23	21
20-29	19	9	9	23	15	9
30-39	11	4	2	7	4	4
40-49	10	1	0	6	3	4
50-	4	5	2	5	3	2
Unknown	2	0	0	1	0	1
Average	23.9	23.9	17.4	20.8	21.1	19.8
Sex; n (%)						
Male	41	12	23	34	33	24
Female	48	21	17	59	23	30
Unknown	0	0	0	1	1	0
Pretreatment; n (%)						
Yes	3	3	0	12	3	6
No	85	31	40	82	54	48
Parasitemia; (%)						
Median (IQR*)	0.14% (0.14%, 0.43%)	0.12% (0.02%, 1.05%)	0.39% (0.11%, 0.79%)	0.32% (0.1%, 0.90%)	0.12% (0.02%, 0.58%)	0.09% (0.02%, 0.41%)

* Interquartile range
