# Supplementary material for: Lack of significant recovery of chloroquine sensitivity in Plasmodium falciparum parasites following discontinuance of chloroquine use in Papua New Guinea
Source: Malar J. 2018 Nov 26;17:434. doi: 10.1186/s12936-018-2585-x (PMC6260888; doi:10.1186/s12936-018-2585-x)
Supplement: Supplementary file 2 — Additional file 2. Allele prevalence in pfcrt and pfmdr1. [file 12936_2018_2585_MOESM2_ESM.pdf]

Additional file 2. Allele prevalence in *pfcrt* and *pfmdr1*

	2016		2017		2018	
	No	Prevalence	No	Prevalence	No	Prevalence
Pfcrt*						
CVMNK	2	0.02	12	0.10	12	0.12
SVMNT	106	0.98	101	0.87	90	0.88
CVMNK+SVMNT	0	0.00	3	0.03	0	0.00
Pfmdr1						
N86	66	0.59	85	0.70	69	0.72
N86Y	44	0.40	33	0.27	27	0.28
N86+N86Y	1	0.01	3	0.02	0	0.00
Y184	89	0.80	103	0.85	86	0.86
Y184F	21	0.19	15	0.12	14	0.14
Y184+Y184F	1	0.01	3	0.02	0	0.00
S1034	80	1.00	88	1.00	100	1.00
S1034C	0	0.00	0	0.00	0	0.00
S1034+S1034C	0	0.00	0	0.00	0	0.00
N1042	67	0.91	78	0.87	89	0.89
N1042D	7	0.09	10	0.11	11	0.11
N1042+N1042D	0	0.00	2	0.02	0	0.00
D1246	95	1.00	115	1.00	100	1.00
D1246Y	0	0.00	0	0.00	0	0.00
D1246+D1246Y	0	0.00	0	0.00	0	0.00

* Amino acids at positions 72–76, mutation underlined
