# Supplementary material for: Lack of significant recovery of chloroquine sensitivity in Plasmodium falciparum parasites following discontinuance of chloroquine use in Papua New Guinea
Source: Malar J. 2018 Nov 26;17:434. doi: 10.1186/s12936-018-2585-x (PMC6260888; doi:10.1186/s12936-018-2585-x)

Additional file 3. Association between $IC_{50}$ values for chloroquine and polymorphisms at position 184 in *pfmdr1* in the parasites with the *pfcrt* K76T mutation

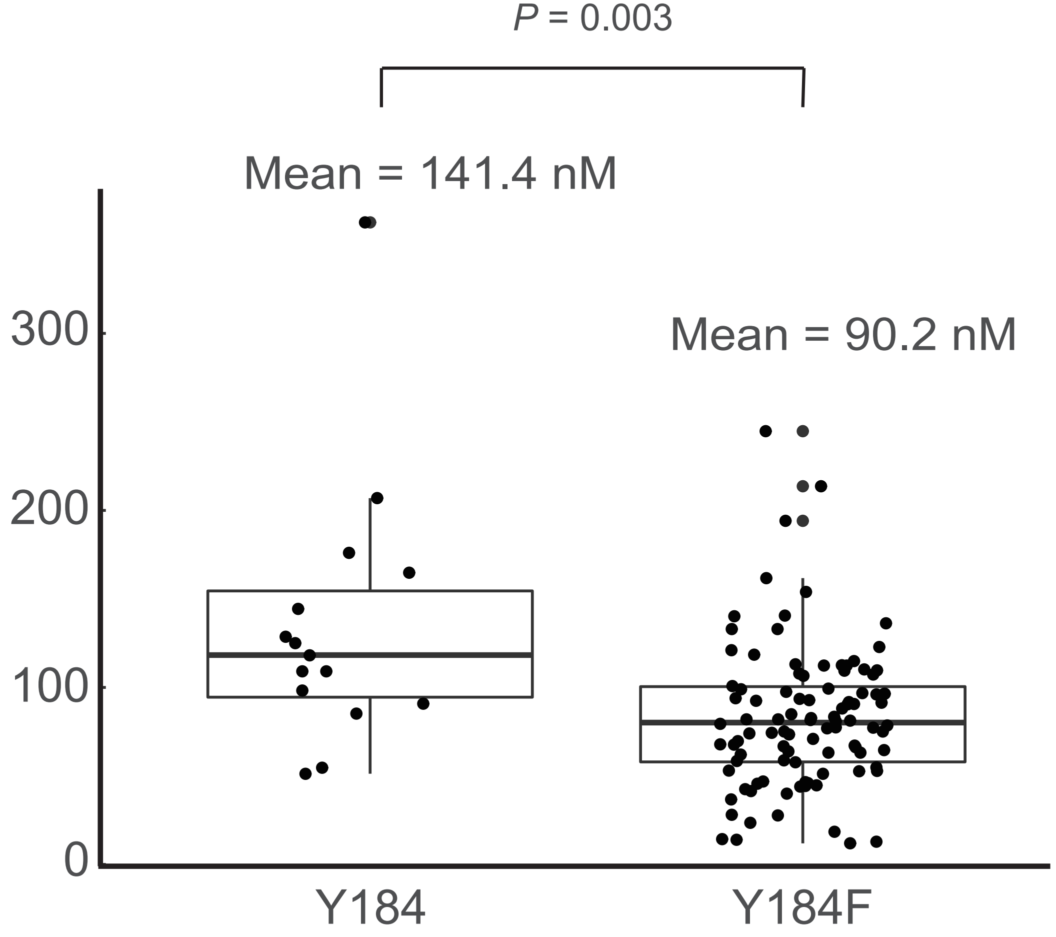

Supplement: Supplementary file 3 — Additional file 3. Association between IC50 values for chloroquine and polymorphisms at position 184 in pfmdr1 in the parasites with the pfcrt K76T mutation. [file 12936_2018_2585_MOESM3_ESM.pdf]
